# Anti‐aging effects of icariin and the underlying mechanisms: A mini‐review

**DOI:** 10.1002/agm2.12284

**Published:** 2024-02-15

**Authors:** Ying Li, Zhi‐Feng Wei, Long Su

**Affiliations:** ^1^ Department of Hematology Changchun Central Hospital Changchun China; ^2^ Department of Hematology The First Hospital of Jilin University Changchun China; ^3^ Jilin Provincial Key Laboratory of Hematology Precision Medicine The First Hospital of Jilin University Changchun China

**Keywords:** aging, anti‐aging effects, icariin, mechanisms, senescence

## Abstract

Aging is an extremely intricate and progressive phenomenon that is implicated in many physiological and pathological conditions. Icariin (ICA) is the main active ingredient of Epimedium and has exhibited multiple bioactivities, such as anti‐tumor, neuroprotective, antioxidant, anti‐inflammatory, and anti‐aging properties. ICA could extend healthspan in both invertebrate and vertebrate models. In this review, the roles of ICA in protection from declined reproductive function, neurodegeneration, osteoporosis, aging intestinal microecology, and senescence of cardiovascular system will be summarized. Furthermore, the underlying mechanisms of ICA‐mediated anti‐aging effects will be introduced. Finally, we will discuss some key aspects that constrain the usage of ICA in clinical practice and the corresponding strategies to solve these issues.

## Graphical abstract

Icariin (ICA) exhibits anti‐aging effects in different physiological and pathological conditions. ICA displays protective effect against reproductive function decline, neurodegenerative diseases, osteoporosis, intestinal senescence, and cardiovascular aging via anti‐inflammation, autophagy induction, antioxidation, maintaining intestinal integrity, and inducing young gut microbiota features.

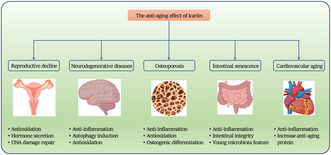

## 1 INTRODUCTION

Aging is an extremely intricate and progressive phenomenon of organisms that is associated with biological age, genetic and epigenetic alterations, and environmental factors.^1^ Cellular aging that is also known as senescence is a hallmark of aging and was first described by Hayflick and Moorhead approximately half a century ago.^2^ Thereafter, cellular senescence was identified as an irreversible cell cycle arrest induced by damage or stress applied to proliferating cells.^3^ Senescent cells remain metabolically active and secrete a range of pro‐inflammatory and proteolytic factors as part of the senescence‐associated secretory phenotype.^4^ The molecular signaling and pathways that regulate cellular senescence, such as p53 pathways, DNA damage signaling, and oxidative stress damage, were well reviewed by Roger et al.^4^ Cellular senescence plays essential roles in blockade of tumorigenesis, tissue damage, and aiding embryonic development. Nevertheless, once excess senescent cells accumulate in tissue during aging, they promote the development of age‐related diseases and limit health lifespan.^3^ There are tremendous enthusiasms to pursue the potential strategies to delay or even reverse the progress of aging or cellular senescence.^5^, ^6^, ^7^ Thus, the field of anti‐aging medicine continues to be one of the research hotspots.

Epimedium is a Chinese medical herb and widely used for sexual enhancement, immunity improvement, anticancer, and anti‐aging treatment.^8^ Icariin (ICA) is the main active ingredient isolated from Epimedium and primarily metabolized into icaritin after digestion that has been approved for treating patients with hepatocellular carcinoma by China Food and Drug Administration (cFDA) in 2022.^9^ Data from in vitro and in vivo studies suggest that ICA has multiple bioactivities, such as anti‐tumor, neuroprotective, antioxidant, hormone regulation, immunological function modulation, and anti‐inflammatory properties.^10^, ^11^, ^12^, ^13^, ^14^, ^15^ Furthermore, ICA exerts a beneficial effect in a variety of age‐dependent diseases.^16^, ^17^, ^18^, ^19^ This review describes the anti‐aging effect of ICA in various invertebrate model organisms and mammals. Meanwhile, the cellular and molecular mechanisms of ICA‐mediated anti‐aging effects will be discussed.

## 2 THE ANTI‐AGING EFFECTS OF ICA AND THEIR MECHANISMS

Extending lifespan has been a beautiful dream of humanity for thousands of years. ICA displayed the ability to extend healthspan in both invertebrate and vertebrate models.^20^, ^21^ ICA and its bioactive form in vivo, icariside II, could extend the lifespan of *Caenorhabditis elegans* with increased thermo and oxidative stress tolerance, slowed locomotion decline, and delayed the onset of paralysis mediated by polyQ and Aβ (1–42) proteotoxicity, which was proved to be dependent on the insulin/insulin growth factor‐1 (IGF‐1) signaling.^20^ ICA also prolonged healthspan and boosted healthy features of mice identified by behavioral tests and bone density analysis. Mechanistically, reduction in oxidative stress and maintenance of the genomic stability by ICA were recognized as two major contributors to such beneficial effect.^21^ These results indicate that ICA is a promising agent for anti‐aging.

### 2.1 Improvement of reproductive function decline

ICA could protect porcine oocytes against damage during aging in vitro via reducing the activity of reactive oxygen species (ROS) and increasing the expression of antioxidant genes (superoxide dismutase 1/2, peroxiredoxin 5, and nuclear factor erythroid 2‐like 2 [Nrf2]). Moreover, ICA treatment not only prevented the oocytes from apoptosis, defects in spindle formation, and chromosomal alignment, but also upregulated the expression of cytoplasmic maturation factor genes (bone morphogenetic protein 15, cyclin B1, MOS proto‐oncogene, serine/threonine kinase, and growth differentiation factor‐9).^12^, ^22^ ICA exhibits protective effects against premature ovarian failure (POF).^23^ In POF animal models induced by d‐galactose, ICA treatment promoted ovary/body weight, ovarian follicular development, and fertility outcomes accompanied by downregulation of follicle stimulating hormone (FSH) and luteinizing hormone (LH) expression and upregulated levels of estradiol and anti‐Müllerian hormone.^16^, ^23^ ICA protected ovarian granulosa cells from d‐galactose‐induced aging with increased cell viability and lower endogenous β‐galactosidase activity via altering γH2AX and 53BP1 expression. These results demonstrated that ICA effectively attenuated ovarian injury via promoting DNA damage repair and restored ovarian function of aging mice to enhance their fertility.^23^ Sertoli cells play crucial roles in spermatogenesis and are impaired by aging. Dietary administration of ICA significantly ameliorated the age‐related decline in testicular function by increasing weights of testicular and epididymal, sperm count and viability, levels of testicular testosterone and estradiol, and improving seminiferous tubule morphology.^24^ ICA protected age‐related Sertoli cells from injury via upregulation of estrogen receptor α (Erα) and Nrf2 signaling.^24^ Thus, ICA may represent a promising agent for the prevention of age‐related ovarian or testicular dysfunction and a potential candidate to improve assisted reproductive technologies.

### 2.2 Protection from age‐related neurodegenerative disorders

Aging is the primary risk factor for most neurodegenerative disorders, including Alzheimer disease (AD) and Parkinson disease (PD). There are few or no effective treatments available for age‐related neurodegenerative diseases, which tend to progress in an irreversible manner and are related to large socioeconomic and personal costs.^25^

One of the characteristics of neurodegenerative events is the impairment of the inflammatory response. Neuroinflammation plays an important role in onset and progression of neurodegenerative diseases.^26^ Microglia‐mediated neuroinflammation importantly contributes to the pathogenesis of neurodegenerative diseases. ICA repressed lipopolysaccharide (LPS)‐induced microglial pro‐inflammatory factor (nitric oxide [NO], IL‐18, and IL‐1β) production via activation of Nrf2 signaling.^27^ In 6‐hydroxydopamine (6‐OHDA)‐induced PD mouse model, ICA attenuated 6‐OHDA‐induced dopamine neurotoxicity and neuronal damage and glial cells‐mediated neuroinflammatory response via activation of Nrf2 signaling pathway.^28^, ^29^ In vitro data showed that ICA protected dopamine neurons from LPS/6‐OHDA‐induced neuronal damage and suppressed microglia activation and pro‐inflammatory factor (TNF‐α, IL‐1β, and NO) production via inhibiting nuclear factor‐κB (NF‐κB) signaling pathway activation. In vivo results showed that ICA remarkedly reduced microglia activation and alleviated LPS/6‐OHDA‐induced neuronal loss and subsequent animal behavior changes.^30^ In one study, the investigators assessed the immunotherapeutic potential of ICA on AD, significantly improved spatial learning and memory retention of mice were observed after ICA treatment. Long‐term application of ICA (oral administration for 8 months) could also reduce hippocampus Aβ deposition and modulate the differentiation of CD4^+^ T cells and release of inflammatory cytokines (TNF‐α, IFN‐γ, monocyte chemoattractant protein‐1 [MCP‐1], IL‐17A, and granulocyte‐macrophage colony‐stimulating factor [GM‐CSF]).^31^ Thus, inhibition of neuroinflammation is an important route for ICA to exert its neuroprotective effects.

Autophagy is the major intracellular machinery for degrading aggregated proteins and damaged organelles, which has been reported to be involved in the occurrence of pathological changes in many neurodegenerative disorders, including AD, PD, Huntington's disease, and amyotrophic lateral sclerosis.^32^ The senescence‐accelerated mouse‐prone 8 (SAMP8) model characterized by early onset and rapid advancement of senescence has been utilized to investigate age‐related neurodegenerative changes associated with aging.^33^ SAMP8 mice treated with ICA showed a robust improvement in spatial learning and memory function along with reduced number of senescent cells and neuronal loss in the brain, and reversed neuronal structural changes in the hippocampi through regulating the expression of autophagy‐related proteins LC3‐II and p62.^34^ Moreover, increased monoamines levels, inhibition of oxidative damage, and decreased acetylcholinesterase activity also participated in ICA‐mediated neuroprotective effects.^33^ In aging rats, ICA treatment improved neuronal degeneration associated with aging via upregulating autophagy‐related proteins light chain 3B (LC3B), Beclin 1, and p‐AMPK in the cortex and hippocampus.^18^ The accumulation of damaged mitochondria and mitophagy are hallmarks of aging and age‐related neurodegeneration. Autophagy/mitophagy is impaired in the hippocampus of APP/PS1 mice (AD) and in Aβ1‐42‐induced PC12 cell models. The combination of β‐Asarone and ICA inhibited cell and mitochondrial damage by inducing autophagy/mitophagy.^35^ Therefore, induction of autophagy is another aspect for ICA‐mediated protection from neurodegeneration.

There are also other mechanisms for ICA to function its bioactivities. In murine model of AD, ICA could improve the neurobehavioral, memory, and motor abilities of the mice via lowering the ferroptosis level and enhancing the resistance to oxidative stress by inhibition of murine double minute 2 (MDM2).^36^ ICA improved spatial learning and memory function determined by the Morris water mazing tasks in aging rats through activation of quiescent neural stem cells.^37^ Both ICA and icaritin exerted regulatory effects in astrocytes under basal conditions and after an inflammatory challenge via IGF‐1 receptor signaling pathway.^38^

Taken together, results of these studies demonstrate that ICA exert a potently protective effect on neurodegeneration. The main mechanisms involved are suppression of neuroinflammation and induction of autophagy. Besides, inhibition of ferroptosis and activation of neural stem cells are also involved in ICA‐mediated neuroprotection.

### 2.3 Improving aging of bone metabolism

The pace of bone formation slows down with aging, which leads to the development of osteoporosis, a common metabolic bone disease. The incidence of osteoporosis has become a public health problem of worldwide concern with the aging of the world's population.^39^ However, the therapeutic strategy for osteoporosis is limited, and ICA may be a promising treatment option for osteoporosis.^40^

ICA is insoluble in water, and platelet‐rich plasm (PRP) was used as a carrier for ICA. In a rabbit model of tendon–bone interface injuries, ICA/PRP treatment was associated with larger cartilaginous metaplasia, increased new bone formation, well‐organized collagen fibers, and robust production of proteoglycans in the regenerated fibrocartilage.^41^ Senescent macrophages (S‐MΦs) present in the bone marrow produce numerous inflammatory cytokines that contribute to the development of osteoporosis. ICA exerted a significant anti‐inflammation effect on S‐MΦs to alleviate bone loss in osteoporotic mice via activating autophagy.^42^ Viability of cultured rat calvarial osteoblasts decreased in hypoxic conditions, which could be reserved by ICA. ICA produced its via reducing production of ROS and malondialdehyde, increasing superoxide dismutase activity, arresting the cell cycle and inhibiting apoptosis. Furthermore, ICA also preserved osteogenic differentiation potential of the hypoxic cells by increased levels of RUNX‐2, OSX, and BMP‐2 gene expression, alkaline phosphatase activity, and formation of mineralized nodules.^43^ Furthermore, ICA exerts its anti‐osteoporotic function by inhibiting JNK/c‐Jun signaling pathway in a bioinformation analysis and verified by in vitro experiments.^40^ In a network pharmacology analysis, ICA occurs to regulate osteoporosis‐related biological processes, cell components, molecular functions, and signaling pathways.^39^ Consequently, the underlying mechanisms of ICA‐mediated anti‐osteoporosis effects include but are not limited to anti‐inflammation, antioxidation, and promotion of osteogenic differentiation.

### 2.4 Improve aging of intestinal tissues

Attenuation of declined gut microbiota of aging mice plays a pivotal role in ICA‐mediated enhancement of youth‐like features.^44^ ICA improved gut microbiota composition of aged mice by reinstating bacteria found in the young mice, while suppressing some bacteria found in the untreated old mice. Direct treatments with ICA and fecal transplant from the ICA‐treated aged mice produce similar anti‐aging phenotypes in the aged mice.^44^ ICA also could maintain intestinal integrity in aging mice. Improvement of motor coordination and learning skills with decreased oxidative stress biomarkers in the serum, brain, kidney, and liver of aged mice were observed after ICA treatment. Additionally, ICA improved the intestinal integrity of the aged mice through upregulating tight junction adhesion molecules and the Paneth and goblet cells accompanied with the reduction of inducible nitric oxide synthase (iNOS) and pro‐inflammatory cytokines (IL‐1β, TNF‐α, IL‐2 and IL‐6, and IL‐12).^44^ Inflammatory cytokines including TNF‐α and IL‐1β impair intestinal barrier function in aging by disrupting intestinal tight junction integrity.^45^ In another study, expressions of inflammatory cytokines were also reduced after ICA intervention, but tight junctions and antioxidant enzymes in aging rats were upregulated.^45^ Meanwhile, ICA treatment also remarkedly upregulated aging‐related signaling molecules, Sirt 1/3/6 6, Pot1α, BUB1 mitotic checkpoint serine/threonine kinase B (BUB1b), FOXO1, E1A‐binding protein p300 (Ep300), annexin A3 (ANXA3), calcium binding protein 1 (Calb1), synaptosomal‐associated protein 25 kDa (SNAP25), and brain‐derived neurotrophic factor (BDNF) in old mice.^44^ Thus, ICA exerts its anti‐aging effects through improving declined gut microbiota, maintaining intestinal integrity, and repressing inflammation in intestinal tissues.

### 2.5 Improving aging of cardiovascular system

Endothelial cellular senescence may cause some cardiovascular disorders.^46^ Homocysteine significantly increased cellular senescence both in vitro and in vivo that could be reserved by ICA, and activation of PI3K/Akt‐epithelial nitric oxide synthase (eNOS)‐dependent signaling pathway may be responsible for this effect of ICA.^47^ ICA could have a protective effect on cardiac aging through upregulating the expression of histone deacetylase SIRT6 and had an inhibitory effect on NF‐κB inflammatory signaling pathways as shown by decreasing mRNA levels of the NF‐κB downstream target genes TNF‐α, intercellular adhesion molecule 1 (ICAM‐1), IL‐2, and IL‐6.^48^ Therefore, activation of PI3K/Akt‐eNOS signaling while inhibition of NF‐κB inflammatory signaling is necessary for the anti‐aging effects of ICA against cardiovascular disorders.

### 2.6 Other aspects

Benign prostatic hyperplasia (BPH) is a common condition in aging men that is frequently associated with troublesome lower urinary tract symptoms.^49^ ICA protected against metabolic syndrome induced BPH in terms of decreased weight and index of the prostate and altered histopathology in a rat model. The underlying mechanisms included the antiproliferative, proapoptotic, antioxidant, and anti‐inflammatory activities of ICA.^50^

## 3 SUMMARY AND FUTURE DIRECTION

Current investigations have demonstrated that ICA displays relatively extensive anti‐aging effects in different age‐related physiological and pathological conditions. ICA effectively protects from declined reproductive function, neurodegeneration, osteoporosis, aging intestinal microecology, and senescence of cardiovascular system. Anti‐inflammation, antioxidation, induction of autophagy, and modulation of cell differentiation are potential mechanisms for ICA to function its bioactivities. Hence, ICA is a promising agent to be used in many age‐related diseases. However, some aspects should be considered before translating ICA into clinical practice. First, ICA is a water‐insoluble component, and exploration of optimal drug dosage form is necessary for clinical usage. Although different drug delivery systems have been investigated in preclinical studies to improve the bioavailability and enhance the therapeutic efficacy of ICA, none of them is tested in clinical settings.^51^, ^52^ Second, ICA has shown the ability to suppress immune responses in several animal models.^10^, ^11^ Administrating ICA to clinical patients should be cautioned as suppression of immune responses could increase the risk for subsequent infection complications, especially for aging patients. Finally, the major metabolic substance of ICA is icaritin, which is approved for usage in clinic in China. It may be an alternative strategy to assess the anti‐aging effects of icaritin in clinical trial. In conclusion, the current research results suggest that ICA is a potential drug for many age‐related diseases although some issues should be addressed to take advantage of its efficacy and simultaneously decrease its side effects.

## AUTHOR CONTRIBUTIONS

Conceptualization: Y.L. and L.S.; Methodology: Y.L., Z‐F.W., and L.S.; Investigation: Y.L., Z‐F.W., and L.S.; Visualization: Y.L., and L.S.; Funding acquisition: L.S.; Project administration: L.S.; Supervision: L.S.; Writing—original draft: Y.L. and L.S.; Writing—review, and editing: all authors.

## FUNDING INFORMATION

This work was supported by grants from Bethune Plan Project (2022B17) and Technology Development Project of Jilin Province (YDZJ202301ZYTS501).

## CONFLICT OF INTEREST STATEMENT

The authors have declared that no conflict of interest exists.

## Data Availability

All data generated or analyzed during this study are included in this article. Further enquiries can be directed to the corresponding author.
